# Bacterial synthesis of PbS nanocrystallites in one-step with l-cysteine serving as both sulfur source and capping ligand

**DOI:** 10.1038/s41598-020-80450-7

**Published:** 2021-01-13

**Authors:** Shiping Wei, Ce Guo, Lijuan Wang, Jiangfeng Xu, Hailiang Dong

**Affiliations:** 1 State Key Laboratory of Biogeology and Environmental Geology, China University of Geosciences, Beijing, 100083 China; 2 School of Marine Sciences, China University of Geosciences, Beijing, 100083 China; 3 School of Materials Science and Technology, China University of Geosciences, Beijing, 100083 China

**Keywords:** Microbiology, Materials science, Nanoscience and technology

## Abstract

The green bacterial biosynthesis of lead sulfide nanocrystallites by l-cysteine-desulfurizing bacterium *Lysinibacillus sphaericus* SH72 was demonstrated in this work. Nanocrystals formed by this bacterial method were characterized using the mineralogical and morphological approaches. The results revealed that the microbially synthesized PbS nanocrystals assume a cubic structure, and are often aggregated as spheroids of about 105 nm in size. These spheroids are composed of numerous nanoparticles with diameter 5–10 nm. Surface characterization of the bacterial nanoparticles with FTIR spectroscopy shows that the l-cysteine coats the surface of PbS nanoparticle as a stabilizing ligand. The optical features of the PbS nanocrystallites were assessed by UV–Vis spectroscopy and PL spectroscopy. The maximum absorption wavelength of the bacterial PbS particles occurs at 240 nm, and the photoluminescence emission band ranges from 375 to 550 nm. The band gap energy is calculated to be 4.36 eV, compared to 0.41 eV for the naturally occurring bulk PbS, with this clear blue shift attributable to the quantum size effect.

## Introduction

Nanocrystalline materials refer to solid materials with crystal grain sizes in the range of 1–100 nm. Compared with their naturally occurring bulk materials, the surfaces of nanograins exhibit comparatively high energy arising from the large volume fraction of molecules and atoms residing in the grain boundaries. These properties confer special electrical and optical attributes to those materials^1,2^, imparting great potential industrial applications to the nanocrystalline materials^3,4^. Lead sulfide (PbS) is a typical IV–VI group semiconductor material. It has a sizable exciton Bohr radius (18 nm) and a rather narrow bulk band gap (0.41 eV). These properties gave PbS a good quantum confinement in the nano-sized structures^5^, thus providing promising potential applications in infrared detectors^6^, solar cells^7^, gas and biosensors^8,9^, and photonic crystals^10^.

Currently, successful synthesis of PbS nanoparticles has utilized three different strategies: physical, chemical or biological approaches^4,11,12^. Generally, metallic Pb^13^, PbO^14,15^, PbNO_3_^5^ and (CH_3_COO)_2_Pb^16^ can act as the lead sources to synthesize PbS nanoparticles, while the sulfur sources can be provided by H_2_S^13^, Na_2_S^15^, Na_2_S_2_O_3_^16^, cysteine^17^, thiourea^5^ and (TMS)_2_S^14^. The physical and chemical approaches towards PbS nanoparticle production are superior in production rate and capacity^16,18,19^. To synthesize different shapes of nanosized PbS particles, various ligands serve as the stabilizers to coat the surfaces of PbS nanoparticles1^1,20,21^. The nature of capping ligands can markedly influence the electro-optical properties of PbS nanoparticles^22^. However, the physical and chemical approaches require a high capital cost, large energy consumption, and usually generate hazardous wastes leading to pollution issues^4,23^. Moreover, the nanoparticles synthesized by those two approaches are regarded as less biocompatible, resulting in their limitations towards biological and medical applications^4,24^.

Considering the drawbacks of the physical and chemical synthesis approaches, recently-developed microbial synthesis of the nanoparticles has attracted interest in the nanoscience and nanotechnology communities and now is preferred approach^25–29^. Previous investigations have demonstrated that heavy metal tolerant microorganisms can induce nanoparticle formation either extracellularly or intracellularly^4,30–32^. Different microorganisms use different mechanisms to synthesize nanoparticles^4,23,33^. In this study, an l-cysteine desulfurizing bacterium, was used to synthesize PbS nanoparticles. *Lysinibacillus sphaericus* SH72 was previously having been shown to produce cysteine desulfhydrase to catalyze the conversion of l-cysteine to pyruvate, hydrogen sulfide and ammonia^34^. During this synthesis process, biogenic hydrogen sulfide immediately reacts with Pb^2+^ to form PbS in the liquid medium, which obviates the danger of toxic H_2_S release to the environment. Moreover, l-cysteine not only serves as the sulfur source for the PbS nanoparticle formation but also acts as a stabilizer to coat the PbS nanoparticle surface, achieving a one-step green synthesis of nanocrystallites of PbS.

## Materials and methods

### Bacterial strain and media

A strain of l-cysteine desulfurizing bacteria *Lysinibacillus sphaericus* SH72 was previously isolated from marine sediments from Beidaihe, China^34^. The medium was used for bacterial synthesis of PbS particles by *L. sphaericus* SH72 was made with (in g/L) containing yeast extract 3.0, beef extract 5.0, peptone 5.0, and NaCl 2.0, with pH adjusted to 7.2 by NaOH/HCl. After autoclaving for 30 min at 121℃, the medium was then supplemented with the filter sterilized l-cysteine 2.0 g/L and (CH_3_COO)_2_Pb·3H_2_O 0.2 g/L.

### Bacterial synthesis and collection of PbS nanoparticles

*L. sphaericus* SH72 was inoculated into 150 mL of sterilized liquid medium held in 500 mL Erlenmeyer flasks. The flask was sealed with a cotton plug and incubated at 28℃, with agitation provided by a rotary shaker at 120 rpm. The culture was incubated with continuous shaking for 5 d to allow PbS nanoparticle formation. The PbS nanoparticles were collected by centrifugation at 5000 rpm for 15 min and then underwent three washes with chloroform to remove the bacterial cell debris, followed by three thorough washes with distilled water. The purified nanoparticles were then air dried for further analyses.

### Characterization

Phase structure of the purified nanoparticles was characterized with a Bruker D2 PHASER X-ray diffractometer (XRD) using 30 kV, 10 mA and scanned from 2θ of 10 to 80, with a scanning rate of 0.02°/s. Morphological observations of the nanoparticles were performed with an FEI Quanta 200F scanning electron microscope (SEM). For transmission (TEM) and high-resolution transmission electron microscopy (HRTEM) observations, the purified bacterial nanoparticles were suspended in water. After ultrasonication, the aqueous suspensions of bacterial nanoparticles were dropped on a carbon-coated copper grid until the solvent evolved was completely dried. The copper grid was then mounted in a TEM-FEI tecnai-F20 instrument, coupled with an Oxford energy-dispersive X-ray spectrometer (EDXS) for chemical analysis. TEM micrograph images were taken and SAED (selected area electron diffraction) patterns were acquired under a 200 kV accelerating voltage. The bacterial nanoparticle surface was characterized by Fourier transform infrared spectroscopy (FTIR, Nicolet iS10 FTIR). The FTIR spectrum was obtained with a resolution of 4 cm^-1^ in the 4000–400 cm^-1^ region. The optical properties of the PbS nanocrystallites were measured on a Hitachi U3900 UV–Vis spectrophotometer from 150 to 800 nm, and an F-4600 FL photoluminescence spectrophotometer.

## Results and discussion

### Structural and morphological characteristics of the bacterial PbS nanocrystallites

**Figure 1:**
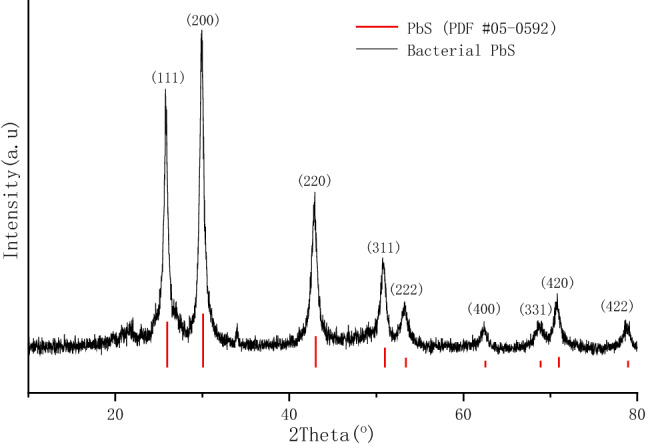
XRD diffraction pattern of bacterial PbS formed by *L. sphaericus* SH72.

The XRD pattern of the bacterial PbS nanoparticles is depicted in Fig. 1. The diffraction peaks at 2θ angles of 25.78, 29.90, 42.92, 50.73, 53.38, 62.37, 68.61, 70.60 and 78.81 degrees index to a cubic PbS mineral phase (JCPDS #05–0592). The peak broadening is indicative of smaller dimensions in nanoparticles. The average crystallite sizes are calculated to be 9.2 nm according to Scherrer’s equation^35^. Previous study showed that *L. sphaericus* SH72 is an l-cysteine desulfurizing bacterium. It can produce cysteine desulfhydrase, by which to catalyze the conversion of l-cysteine to pyruvate, hydrogen sulfide and ammonia^34^. The hydrogen sulfide released from l-cysteine immediately reacts with Pb^2+^ to form PbS mineral in the liquid medium.

**Figure 2:**
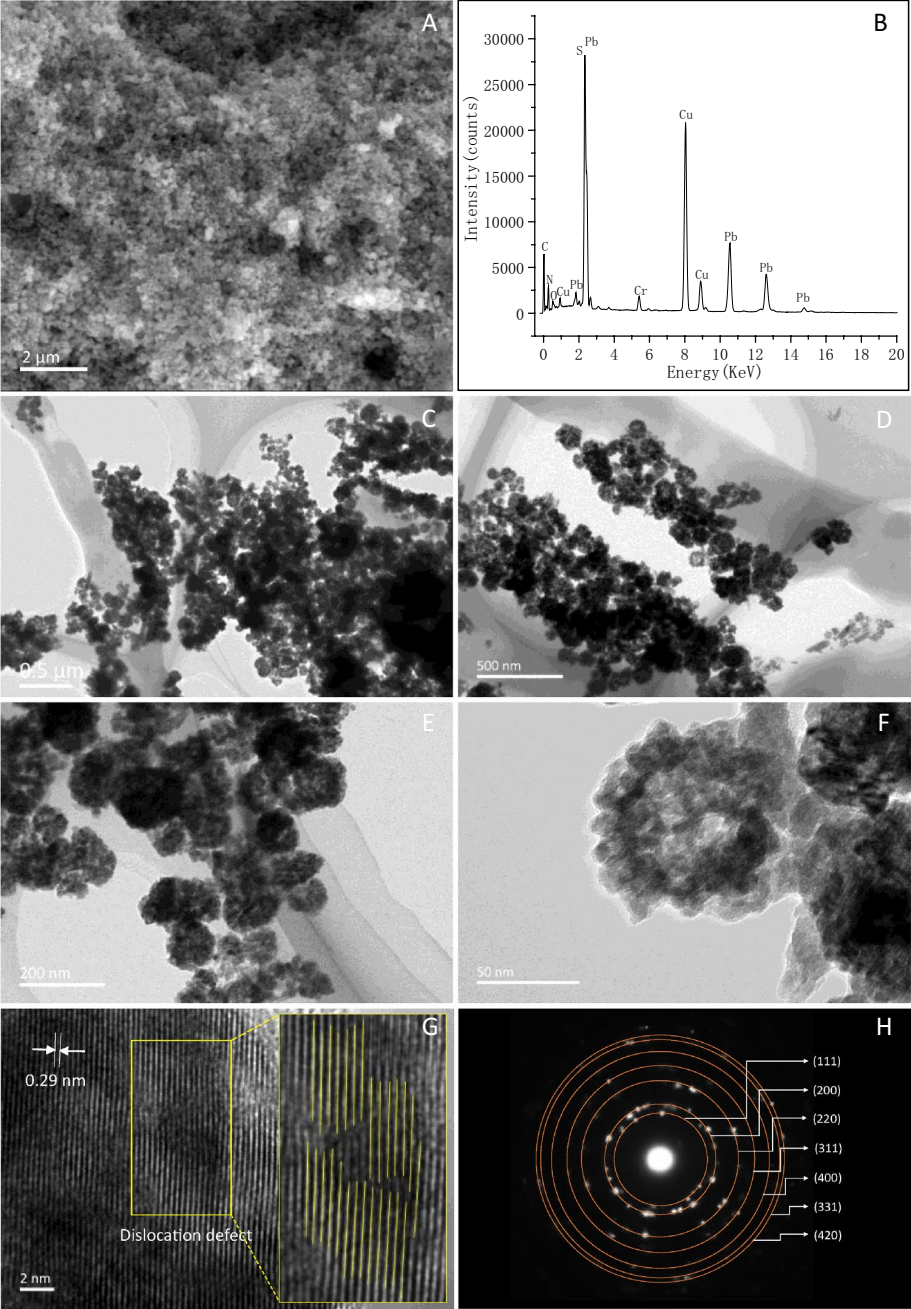
SEM (**A**), EDXS (**B**), TEM (**C**–**F**), HRTEM (**G**) and SAED (**H**) characterizations of the bacterial PbS nanoparticles. The Cu and Cr peaks in (**B**) came from the TEM grid. The yellow rectangle zones in (**G**) show a linear dislocation defect in the bacterial PbS nanocrystal.

The SEM image shows that the particles occur as spheroids (Fig. 2A). The EDXS spectrum shows that the chemical composition of the bacterial particles is mainly composed of Pb and S, which yields a Pb:S ratio of about 0.9:1 (Fig. 2B). The minor elements of C, N and O are also present in the bacterial particles, suggesting that certain organic molecules probably coat the bacterial PbS nanoparticles^11,21^.

Upon a closer examination of the spheroids by TEM (Fig. 2C–F), the spheroids are present as globular aggregates of about 105 nm in size (Fig. 2C–E), which consist of numerous nanoparticles ranging in size 5–10 nm (Fig. 2E). The spherically shaped aggregates of the PbS particles synthesized by *L. sphaericus* SH72 are apparently different from those PbS particles synthesized by yeasts *Rhodosporidium diobovatum*, *Torulopsis* sp and the bacterium *Rhodobacter sphaeroides*^26,30,36^, in which the PbS particles are virtually monodispersed spherical nanoparticles. However, previous reports have shown that the morphology of chemically synthesized ZnS particles with cysteine caps is unaggregated and has a uniform shape and size^37^.

HRTEM and SAED analyses were performed to confirm the observed XRD results (Fig. 2G,H). The HRTEM micrographs of an individual bacterial PbS nanocrystal show a lattice spacing of 0.29 nm, well matched with the (200) planes of a cubic PbS structure (Fig. 2G)^35,36^, and the formation of a linear dislocation defect went through the crystal was also observed in the bacterial PbS nanocrystal (Fig. 2G). The corresponding SAED pattern shows a set of discontinuous spots, forming concentric rings (Fig. 2H) that indicate a random orientation of the PbS nanocrystals^15,19^. The seven rings can be ascribed to Miller indices of the cubic PbS phase (111), (200), (220), (311), (400), (331), and (420) (Fig. 2H). The HRTEM and SAED analyses on the structure of the bacterial PbS nanocrystals are congruent with the XRD data.

### Optical properties of the bacterial PbS nanocrystallites

**Figure 3:**
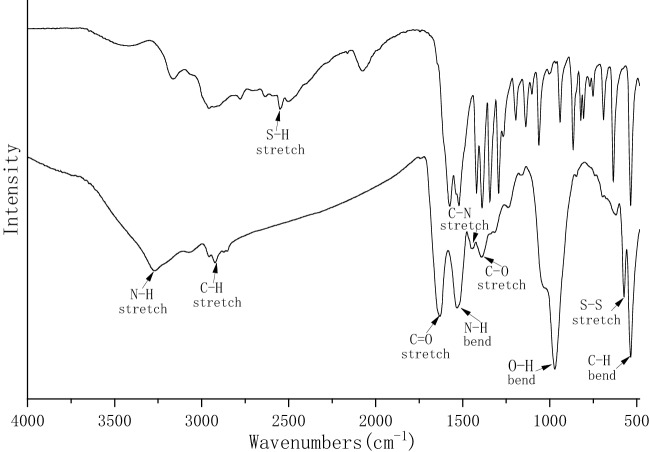
FTIR spectra recorded for l-cysteine (top) and the bacterial PbS nanoparticles (bottom).

FTIR spectroscopic measurement was performed to reveal the bacterial PbS nanoparticles’ surface coating of organic molecules (Fig. 3). It gives two absorption peaks at 3269 cm^-1^ and 2923 cm^-1^, which arise from the N–H and C–H stretching vibrations, respectively. The absorption peaks at 1631 cm^-1^, 1442 cm^-1^, and 1392 cm^-1^, are attributed to C=O, C-N, and C-O stretching vibrations, respectively. A bending vibration of N–H was observed at 1532 cm^-1^. The 573 cm^-1^ peak shows a vibration stretching of an S–S bond. The FTIR spectrum of l-cysteine shows a typical S–H vibrational band appearing at 2555 cm^-1^. However, this was not observed once cysteine is associated with the bacterial nanoparticles, indicating cleavage of the S–H bond. Instead, S–S bond formation was observed between the l-cysteine molecules and the PbS nanoparticles. Previous studies have shown that cysteine could bind to PbS, ZnS and silver nanoparticles through a thiolate linkage^20,37–39^, serving as the stabilizing ligand to suppress the agglomeration of numerous particles^11,21^.

**Figure 4:**
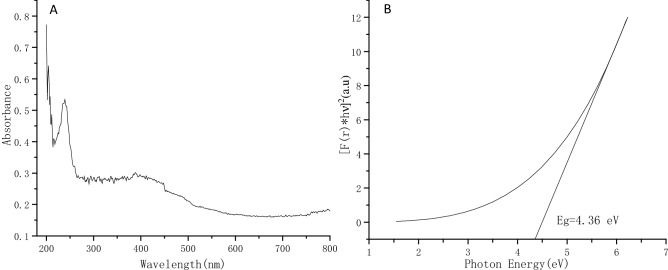
UV–Vis spectrum (**A**) and band gap plot (**B**) of the bacterial PbS nanoparticles.

The UV–Visible spectrum of the bacterial PbS nanoparticles shows that a peak at 205–250 nm was observed (Fig. 4). Compared with the absorption coefficient of the bulk PbS at 3020 nm^40^, the bacterial PbS nanoparticles exhibit a significant blue shift. This is indicative of distinct quantum confinement arising from the reduction in particle dimensions. The optical band gap (E_g_) of the bacterial PbS nanoparticles is calculated according to the equation of αhγ = A(hγ-E_g_)^1/2^ (α is absorption coefficient, h is Plank’s constant, γ is the incident photon frequency, A is absorbance), the result indicates that band gap of the bacterial nanoparticles is 4.36 eV, demonstrating a distinct 0.41 eV blue shift of the band gap for the naturally occurring PbS^35^.

**Figure 5:**
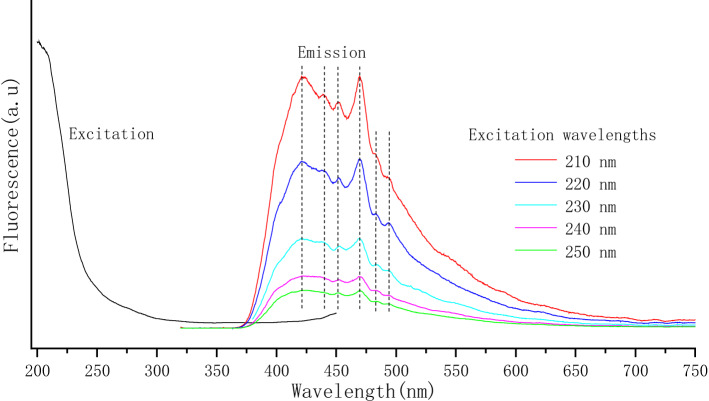
Photoluminescence (PL) excitation and emission spectra of the bacterial PbS nanoparticles.

Figure 5 shows the excitation and photoluminescence (PL) spectra of the bacterial PbS nanoparticles. It is observed that the emission bands occur over a broad wavelength range of 375–550 nm, and the emission peaks of the as-prepared bacterial PbS nanoparticles remain stable under excitation at different wavelengths. To further investigate the stability of the bacterial PbS nanoparticles, a storage of bacterial PbS nanoparticles for eight months were performed UV–Vis and PL spectra analyses, it shows that the corresponding absorbance spectra peak maxima wavelength and intensity have not been changed. Previous studies have shown that the defect-related luminescence often occurs in the inorganic crystals^41–44^. As Fig. 2G shows, a linear dislocation defect was observed in the bacterial PbS nanocrystals, which is inferred for the mechanism of luminescence. However, the process and mechanism by which dislocations form during the bacterial PbS crystal growth need to be further studied.

## Conclusion

In this study, it has been demonstrated that *L. sphaericus* SH72 could synthesize PbS nanocrystallites with l-cysteine serving as the sulfur source as well as the coating ligand. TEM observation shows that the PbS nanocrystallites exhibit globular aggregations in size of about 105 nm, which consist of numerous nanoparticles with dimentions ranging from 5 to 10 nm. XRD and electron diffraction patterns confirm the bacterial nanocrystals form as cubic structures of PbS. Characterization of the bacterial nanoparticles by FTIR indicates that l-cysteine coats the nanoparticle surfaces as a stabilizing agent. The UV–Vis and PL spectra show a significant blue shift and an increased bandgap as compared to bulk PbS, with the increase attributable to its quantum confinement. This one-step green synthesis of nanocrystallites by an l-cysteine -desulfurizing bacterium has important advantages over other biosynthetic methods and promising potential applications in the area of semiconductor materials.
